# Cardiac Manifestations in Systemic Lupus Erythematosus: Clinical Correlates of Subclinical Echocardiographic Features

**DOI:** 10.1155/2019/2437105

**Published:** 2019-01-10

**Authors:** Alaa A. A. Mohamed, Nevin Hammam, Mona H. EL Zohri, Tamer A. Gheita

**Affiliations:** ^1^Rheumatology, Rehabilitation and Physical Medicine Department, Faculty of Medicine, Assiut University Hospitals, Assiut, Egypt; ^2^Faculty of Medicine & Dentistry, University of Alberta, Edmonton, AB, Canada; ^3^Internal Medicine Department, Faculty of Medicine, Assiut University Hospitals, Assiut, Egypt; ^4^Rheumatology Department, Faculty of Medicine, Cairo University, Giza, Egypt

## Abstract

**Objectives:**

This study aims to correlate subclinical echocardiographic features with the clinical, laboratory, and therapeutic profiles of the patients to characterize risks for systemic lupus erythematosus (SLE) cardiac diseases.

**Methods:**

The study included 59 SLE patients. Demographic data, disease characteristics, and current therapies were recorded, and the anthropometric measurements and routine laboratory tests were performed. The disease activity by the SLE Disease Activity Index-2K (SLEDAI2K) and the presence of metabolic syndrome (MetS) were assessed. Two-dimensional echocardiography was performed.

**Results:**

The mean age of the patients was 31.3 ± 10.5 years, and the disease duration was 5.18 ± 4.1 years. 86.4% of the patients were females. Cardiac presentations by echocardiography were mainly mitral regurgitation (33.9%), tricuspid regurgitation (32.2%), mitral thickening (18.6%), aortic thickening (13.6%), pericardial effusion (13.6%), and pulmonary hypertension (8.5%) in order of frequency. The frequency of different echocardiographic findings with respect to other clinical phenotypes showed peaks with renal disease, MetS, and leukopenia. Components of MetS (triglycerides, high systolic blood pressure) and avascular necrosis were significant predictors for pericardial diseases (OR=1.011 CI 95% 1-1.022, p=0.046, OR=1.157 CI 95% 1.025-1.307, p=0.018, and OR=74.78 CI 95% 2.52-2215.76, p=0.013, respectively), and it is likely that hydroxychloroquine was protective against them. Age of the patients was a significant predictor for tricuspid regurgitation (OR=1.063 CI 95% 1.004-1.126, p=0.036). Mucosal ulcers were negative predictors for mitral thickening and regurgitation (OR=0.2 CI 95% 0.059-0.673, p=0.009). The use of corticosteroids appeared to protect against a number of valve lesions especially tricuspid regurgitation (OR=0.299 CI 95% 0.088-1.019, p=0.054).

**Conclusion:**

This study highlighted different echocardiographic features and identified clinical predictors of different cardiac pathologies aiming to determine patients at risk and improve the prognosis of SLE cardiac diseases.

## 1. Introduction

Systemic lupus erythematosus (SLE) is an autoimmune disease characterized by affection of different organs in the body [1]. The cardiovascular involvement in SLE and the subsequent cardiovascular disease (CVD) predispose to a significant morbidity and can raise the mortality risk [2], which occurs more often late in the disease in the absence of active SLE states [3]. Cardiovascular events are proportionally higher in SLE compared to general populations of comparable age and sex [4]. Traditional cardiovascular risk factors have partially explained the cardiovascular events in SLE [5]. In addition, metabolic syndrome (MetS) has been proven to raise the risk of vascular events and organ damage in SLE [6]. The endothelial damage in SLE is believed to be due to several factors which predispose to premature atherosclerosis with subsequent cardiac events. Older age, smoking status, high C-reactive protein (CRP), and antiphospholipid antibodies (aPLs) were among the factors associated with vascular events [7]. Reduced renal function, high C3, and cumulative steroid use were among SLE-related factors to coronary artery calcification [8], although the use of corticosteroid was not found to be consistently associated with CVDs [9]. From another perspective, atherosclerosis and cardiac diseases in SLE are thought to be attributed to chronic inflammation [10].

Cardiac diseases in lupus may involve the endocardium, myocardium, and pericardium and may be responsible for fatal outcome [11, 12]. Some cardiovascular abnormalities are seen with positive anti Ro/SS-A, anti La/SS-B, anti-cardiolipin (aCL), and anti-double-stranded DNA (anti-dsDNA) [12–14]. However, most of these antibodies have not yet fully explained the pathogenic mechanisms of different SLE cardiac features including different valvular affection [12, 15, 16]. Moreover, endothelial dysfunction was reported in early SLE cases without CVDs which was mostly not related to aCL antibodies, disease activity, or disease duration but rather related to renal disease, diastolic hypertension, and diabetes in SLE [17, 18].

The pathogenic mechanisms of different cardiac diseases in SLE are still incompletely understood. It is not well characterized whether SLE holds a risk of CVDs in general, or CVDs, like other lupus manifestations, represent a phenotype occurring in subgroups of patients.

Thus, this study aimed to correlate the subclinical echocardiographic features of SLE with the clinical, laboratory, and therapeutic profiles of the patients in order to characterize the risks of cardiac diseases in SLE.

## 2. Methods

### 2.1. Patients and Methods

This cross-sectional study was conducted in a 6-month period in the Rheumatology Department of Assiut University Hospitals, Egypt. The study was approved by the ethical committee of Assiut Faculty of Medicine. Patients aged ≥ 18 years and fulfilling Systemic Lupus International Collaborating Clinics (SLICC) classification criteria for SLE [19] were enrolled after being consented. The patients were not known to have any cardiac diseases prior to enrollment.

### 2.2. Clinical Evaluation

Demographic data of patients were collected including age, gender, and smoking status. The disease duration and age at first diagnosis were recorded. The disease activity was evaluated using the SLE Disease Activity Index-2K (SLEDAI-2K) [20]. Current drug administration (dose and duration) was recorded for the patients including corticosteroids (CS), hydroxychloroquine (HCQ), disease modifying anti-rheumatic drugs (DMARDs), anti-hyperlipidemia, and anti-hypertensive therapies. The body mass index was calculated and waist circumference (WC) was assessed. The diagnosis of MetS was determined according to the National Cholesterol Education Program Adult Treatment Panel III (NCEP) [21].

### 2.3. Laboratory Tests

Routine laboratory tests were done including erythrocyte sedimentation rate (ESR) measured by Westergren blot, full blood cell count, fasting blood glucose levels (FBG), liver and kidney function tests, creatinine clearance, 24-hour protein in urine, serum uric acid (SUA), and urine analysis. Autoantibodies such as anti-dsDNA measured by enzyme-linked immunosorbent assay (ELISA) and complement levels (C3 and C4) were measured. Lipid profile markers including total cholesterol (TC), high density lipoprotein (HDL), low density lipoprotein (LDL), and triglycerides (TG) were assessed for all patients.

### 2.4. Echocardiography

Two-dimensional echocardiography was used to assess all patients for cardiac features. Pulmonary arterial hypertension (PAH) was considered when systolic pulmonary artery pressure ≥36 mmHg [22]. Ejection fraction <54% was considered abnormal [23, 24]. Fractional shortening percentages of left ventricle were calculated from end-diastolic and end-systolic dimensions using the formula (*LVEDD* − *LVESD*) ÷ *EDD* × 100, and the values for mild (20-25%), moderate (15-19%), and severe (≤14%) fractional shortening were adopted from the following website: https://web.stanford.edu/group/ccm_echocardio/cgi-bin/mediawiki/index.php/Left_ventricle_size.

### 2.5. Statistical Analysis

Data were subjected to tests of normality, Shapiro-Wilk, and Kolmogorov-Smirnov. Participants' characteristics were presented as means ± standard deviations (SD) and medians (Interquartile ranges) as appropriated (for continuous variables) or as numbers and percentages (for categorical variables). For categorical variables, Chi-Square and Fisher's exact tests were used to report comparisons between the groups as appropriated. Mann-Whitney U test was used for numerical variables to compare between two groups. Correlations were tested using Spearman's rho correlation coefficient. The independent associations of the variables were tested in a multivariate logistic regression analysis when the variables showed a significance level p<0.05 in the univariate analysis or were assumed to have clinical relevance. All regression analyses' results were expressed as odds ratio (OR) and 95% confidence interval (CI). P values less than 0.05 were considered significant. Risk estimates were determined for categorical variables of interest in the cross tabulations and provided in the supplementary data (available here). All statistical analyses were carried out using the statistical program the Statistical Package for Social Science (SPSS) version 24 (SPSS Inc.; Chicago, IL, USA).

## 3. Results

A total of 59 patients with SLE were recruited in this study, and 86.4% of them were females. The demographic, clinical, current therapeutic, and laboratory characteristics are demonstrated in Table 1.

The most frequent cardiac presentations by echocardiography in lupus patients were mitral regurgitation, tricuspid regurgitation, mitral thickening, aortic thickening, pericardial effusion, and PAH, in order of frequency as shown in Table 2.

**Figure 1:**
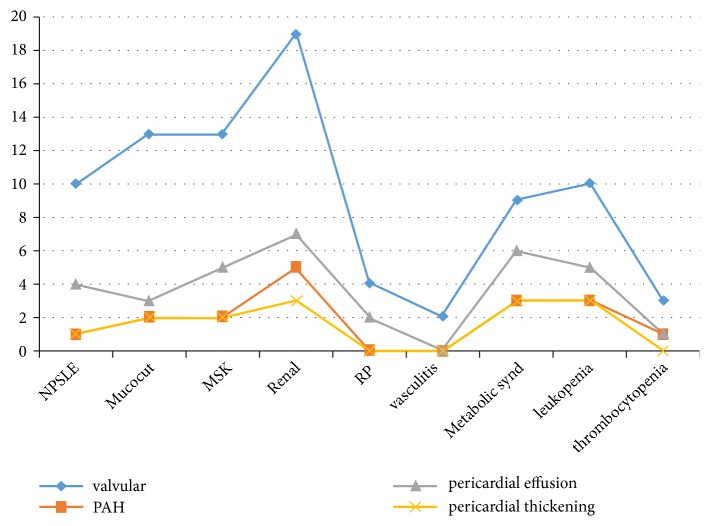
**The frequency of different echocardiographic findings in different lupus phenotypes. ** PAH, pulmonary arterial hypertension; NPSLE, neuropsychiatric systemic lupus erythematosus; Mucocut, mucocutaneous; MSK, musculoskeletal; RP, Raynaud's phenomenon.

**Figure 2:**
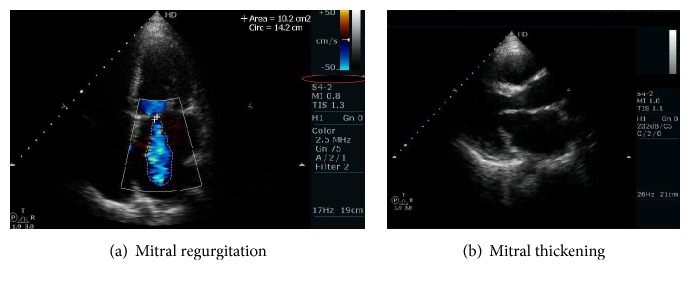
Echocardiography of mitral regurgitation (a) and mitral thickening (b).

The frequency of different echocardiographic findings with respect to other clinical phenotypes showed peaks with renal disease, MetS, and leukopenia with fewer incidences in others as illustrated in Figure 1. The echocardiography of mitral regurgitation and mitral thickening in two of the patients is shown in Figure 2.

There were no significant associations between the echocardiographic features and the SLEDAI scores or ESR. However, studying the association of different demographic, clinical, laboratory, and therapeutic features with the echocardiographic findings revealed a number of significant associations. It was revealed that all patients with pericardial thickening met the diagnosis of MetS and were not on azathioprine (AZA). They had considerably lower cumulative HCQ doses (p=0.042) and HDL levels (p=0.004) but significantly higher TG levels (p=0.024). Patients with pericardial effusion were of significantly older age when diagnosed with SLE (p=0.043) and had higher TG levels (p=0.03), lower cumulative HCQ doses (p=0.018), higher systolic and diastolic blood pressures (p=0.013, 0.034, respectively), and more frequent MetS, avascular necrosis (AVN), and anti-hypertensive medications use (p=0.008, 0.046, 0.053, respectively). Remarkably, all patients with mitral thickening had seizures and showed more frequent cognitive impairment (p=0.033) but less frequent mucosal ulcers and arthritis (p=0.083, 0.079, respectively). Similarly, mitral regurgitation was associated with more frequent cognitive impairment (p=0.014) but less frequent mucocutaneous lesions (p=0.019). It was also noticed that all patients with aortic thickening suffered from seizures, were less CS users (p=0.049), and had significantly lower cumulative CS doses (p=0.019) and HDL levels (p=0.023). Pyuria was the only parameter associated with aortic regurgitation (p=0.035). Tricuspid regurgitation occurred in substantially older patients (p=0.026). The occurrence of malar rash and the use of CS tended to be less frequent in patients with tricuspid regurgitation (p=0.063, 0.053, respectively). Similarly, patients with pulmonary regurgitation did not present with mucocutaneous disease or use CS. Importantly, PAH was seen in patients with significantly lower LDL levels (p=0.046) (details of the previous associations and risk estimates for  different echocardiographic features are provided in the  supplementary data).

Forward logistic regression was performed to detect the independent association of each echocardiographic feature with its demographic, clinical, and laboratory correlates. The independent associations are shown in Table 3.

## 4. Discussion

The cardiac representation as initial manifestations in lupus is uncommon, and cardiac diseases tend to be clinically silent for long periods [13, 25]. Yet, the cardiac complications of lupus are potentially serious. Understanding the pathogenesis of cardiovascular complications is very important and incompletely justified [5, 26]. In the current study, we aimed to elucidate the associations of different subclinical echocardiographic findings with patients' and disease characteristics via screening of 59 SLE patients by echocardiography. Overall, more than half the SLE cohort, in the current work, had a sort of cardiac affection; mitral and tricuspid regurgitation were the most frequent kinds of lesions. Generally, renal affection, MetS, and leukopenia were the phenotypes with frequent cardiac affections. Analysis of individual cardiac involvements with patients' and disease characteristics and therapeutic profiles demonstrated a link between pericardial diseases and blood lipids, blood pressure, MetS, AVN, and proteinuria, while HCQ appeared to be protective against them. Mitral diseases were more common with cognitive impairment whereas they were less common with mucocutaneous diseases. Also, lupus mucocutaneous disease occurred less frequently with tricuspid and pulmonary regurgitation. Administration of CS appeared to be protective against tricuspid and pulmonary regurgitation and aortic thickening.

The frequency of occurrence of echocardiographic abnormalities in this study tends to be similar to other previous observations with the mitral and tricuspid valve involvement being the most frequent, while myocardial dysfunction is less [16, 25]. There are conflicting results regarding the association of disease activity with cardiac diseases. In the study of* Li et al.* [27], PAH was noticed to be associated with disease activity; nevertheless, PAH was more common in those with low SLEDAI and ESR as reported by* Huang et al.* [28]. In another report, only PAH and myocarditis correlated with lupus disease activity, which was not the case with the valvular, pericardial, or coronary artery diseases [29]. This might imply diminished direct links between lupus flares and cardiac involvement. Consistent with our findings, cardiac manifestations were reported to present with renal and hematologic diseases [29].

This study revealed a prominent association of pericardial diseases with hyperlipidemia and high blood pressure. In agreement with that, proteinuria has recently been reported to be a predictor of pericarditis [30]. The risk of AVN with renal disease and hypertension was previously established [31], and it is noticed in the current work that AVN is a predictor of pericardial effusion as well. In this context, the protective effect of HCQ against metabolic disorders in SLE [32, 33] seems to modify the risk of pericardial diseases.

Despite the limited number of cases in the current study, aortic regurgitation appeared to be associated with an element of renal flare. The pathogenic mechanism of aortic valve pathology is poorly understood. Although it was linked to antiphospholipid syndrome [34], severe aortic insufficiency was reported without aPLs [11, 35].

The PAH negatively correlated with LDL levels in the current study. In agreement with this finding,* Kopec et al.* reported recently significantly lower LDL levels in PAH patients, including those with connective tissue diseases, compared to controls, and lower LDL levels were significantly associated with high mortality rate from PAH [36]. Moreover, the reversal of chronic thromboembolic PAH has raised levels of LDL compared to unchanged levels in patients with non-reversed chronic thromboembolic PAH [36]. Unexpectedly, there was no association between the presence of vasculitis or Raynaud's phenomenon and PAH. This is probably due to the low number of patients with these presentations.

Neurological complications such as cognitive impairment and seizures were associated with mitral valve diseases, in the current work. Perhaps aPLs are to be blamed for the development of mitral valve disease and lupus neurologic disease [37, 38]. The link between mitral valve diseases and cognition defects was previously regarded as a causal relationship since mitral valve disease predisposes to reduced cerebral perfusion [39]. Overall mucocutaneous diseases especially mucosal ulcers were negatively correlating with mitral valve diseases in this study. Mucocutaneous disease was also a negative predictor for tricuspid and pulmonary regurgitation. These negative correlations might be attributed to different disease pathologies and warrant further investigations.

In the current study, corticosteroids use was suggested to be protective against a number of valve lesions such as tricuspid regurgitation and aortic thickening and maybe pulmonary regurgitation, which opposes its identified risk of causing harmful events on the blood vessels by inducing the risk factors for atherosclerosis [40]. Previously, the protective anti-inflammatory effect of CS on inhibiting intima proliferation was discussed in [41, 42]. However, data about CS is complex and has never been established.

The limitation of this study was lack of testing different autoantibodies to clearly delineate their coupled risks for different cardiac diseases. Also, our findings need to be strengthened by future larger scale prospective studies.

In conclusion, this study highlighted different echocardiographic features in SLE patients and identified their clinical predictors. The clinical impact of such a study is to identify SLE patients at risk of developing serious cardiac complications via the clinical predictors aiming to improve the prognosis of SLE cardiac diseases.

## Figures and Tables

**Table 1:** Patients' demographics and disease characteristics.

	**Parameters**	**Total No=59**
		No. (%)
**Demographic**	Age/ age at diagnosis (years) #	31.3±10.5 / 27.5± 10.1
	Female gender	51 (86.4%)
	Smoking	2 (3.4%)
	Disease duration (years) #	5.18 ± 4.1

**Clinical profile**	Systolic / Diastolic BP§	120/80 (110-130/70-90)
	(i) Systolic hypertension	3 (5.1%)
	(ii) Diastolic hypertension	21 (35.6%)
	NPSLE	18 (30.5%)
	(i) Seizures	2 (3.4%)
	(ii) Psychosis	2 (3.4%)
	(iii) Headache	11 (18.6%)
	(iv) Cognitive impairment	8 (13.6%)
	Retinal disease	2 (3.4%)
	RP	13 (22%)
	Avascular necrosis	3 (5.1%)
	Vasculitis	6 (10.2%)
	Serositis	7 (11.9%)
	(i) Pleurisy	4 (6.8%)
	(ii) Pleural effusion	2 (3.4%)
	(iii) Pericarditis	2 (3.4%)
	(iv) Pericardial effusion	8 (13.6%)
	Musculoskeletal	30 (50.8%)
	(i) Arthritis	20 (33.9%)
	(ii) Myositis/myalgia	12 (20.3%)
	Mucocutaneous disease	34 (57.6%)
	(i) Malar rash	29 (49.2%)
	(ii) Oral/nasal ulcers	30 (50.8%)
	(iii) Alopecia	16 (27.1%)
	MetS	21 (35.6%)
	Leukopenia	20 (33.9%)
	Thrombocytopenia	7 (11.9%)
	Renal disease	39 (66.1%)
	(i) Proteinuria (>500mg/ 24 h)	31 (52.5%)
	(ii) Casts	10 (16.9%)
	(iii) Hematuria (>5 cells/HPF)	6 (10.2%)
	(iv) Pyuria (>5 cells/HPF)	13 (22%)
	SLEDAI scores §	13 (8-20)

**Laboratory profile**	ESR (1^st^ hour) §	34 (20-73)
	Cholesterol (gm/dl) §	153 (131- 216)
	LDL (gm/dl) §	85 (63.5- 116.5)
	HDL (gm/dl) §	51 (37- 65)
	TG (gm/dl) §	95 (73.5- 150)
	SUA (gm/dl) §	4.1 (3.8- 5.2)

**Therapeutic profile**	MTX use	8 (13.6%)
	HCQ use	52 (88.1%)
	HCQ cumulative dose§	3400 (400-14400)
	AZA use	33 (55.9%)
	CS use	41 (69.5%)
	CS cumulative dose§	180 (20-720)
	CYC use	16 (27.1%)
	Statins use	3 (5.1%)
	LDA use	30 (50.8%)
	Anti- HTN use	24 (40.7%)
	Anti-coagulant use	12 (20.3%)

#, mean ± standard deviation; §, median (interquartile range); BP, blood pressure; RP, Raynaud's phenomenon; NPSLE, neuropsychiatric systemic lupus erythematosus; MetS, metabolic syndrome; SLEDAI, systemic lupus erythematosus disease activity index; s. cholest., serum cholesterol; LDL, low density lipoprotein cholesterol; HDL, high density lipoprotein cholesterol; TG, triglycerides; SUA, serum uric acid; MTX, methotrexate; HCQ, hydroxychloroquine; AZA, azathioprine; CS, corticosteroids; CYC, cyclophosphamide; LDA, low dose aspirin; anti-HTN, anti-hypertensives.

**Table 2:** Different echocardiographic features of SLE patients.

**Echocardiographic features**	**n (**%**)**
Pericardial thickening*∗*	4 (6.8%)

Pericardial effusion*∗*	8 (13.6%)

Overall valve lesions	28 (47.5%)
(i) Mitral thickening	11 (18.6%)
(ii) Mitral stenosis	1 (1.7%)
(iii) Mitral regurgitation	20 (33.9%)
(iv) Aortic thickening	8 (13.6%)
(v) Aortic stenosis	nil
(vi) Aortic regurgitation	4 (6.8%)
(vii) Tricuspid thickening	1 (1.7%)
(viii) Tricuspid stenosis	nil
(ix) Tricuspid regurgitation	19 (32.2%)
(x) Pulmonary thickening	1 (1.7%)
(xi) Pulmonary stenosis	1 (1.7%)
(xii) Pulmonary regurgitation	3 (5.1%)

PAH	5 (8.5%)

LVEF <54%	2 (3.4%)

LV hypokinesia	1 (1.7%)

Fractional shortening (LVEDD-LVESD/LVEDD*∗* 100%)	
(i) mild (20-25%)	1 (1.7%)
(ii) moderate (15-19%)	1 (1.7%)
(iii) severe (≤14%)	nil

Echo free #	26 (44.1%)

*∗*Pericardial thickening almost always presented in the patients with effusion but pericardial effusion presented solely without thickening in four cases. The valve lesions presented in patients in different combinations. PAH, pulmonary arterial hypertension; LVEF, left ventricular ejection fraction; LV, left ventricle; LVEDD, left ventricular end-diastolic dimension; LVESD, left ventricular end-systolic dimension. Echo free # denotes patients without any cardiac signs by echo.

**Table 3:** Independent associations with echocardiographic abnormalities.

**Echocardiographic feature**	**Logistic regression**
Pericardial thickening	**TG **
	OR=1.011 CI 95% 1- 1.022
	p=.046

Pericardial effusion	**Systolic BP **
	OR=1.157 CI 95% 1.025- 1.307
	p=.018
	**AVN **
	OR=74.78 CI 95% 2.52- 2215.76
	p=.013

Mitral thickening	**Mucosal ulcers **
	OR=0.2 CI 95% 0.059-0.673
	p=.009

Mitral regurgitation	**Mucosal ulcers **
	OR=0.2 CI 95% 0.059-0.673
	p=.009

Tricuspid regurgitation	**Current age **
	OR=1.063 CI 95% 1.004-1.126
	p=.036
	**CS **
	OR=0.299 CI 95% 0.088-1.019
	p=.054

TG, triglycerides; BP, blood pressure; AVN, avascular necrosis; CS, corticosteroids.

## Data Availability

The data used to support the findings of this study are included within the article and the supplementary materials.

## Supplementary Materials

Supplementary Materials Table 1: Highlights on the associations of demographics, clinical phenotypes, laboratory profile, and drug therapy with the common echocardiographic features in lupus patients.
