# Supplementary material for: Cardiac Manifestations in Systemic Lupus Erythematosus: Clinical Correlates of Subclinical Echocardiographic Features
Source: Biomed Res Int. 2019 Jan 10;2019:2437105. doi: 10.1155/2019/2437105 (PMC6348873; doi:10.1155/2019/2437105)
Supplement: Supplementary Materials — Table 1: Highlights on the associations of demographics, clinical phenotypes, laboratory profile, and drug therapy with the common echocardiographic features in lupus patients. [file 2437105.f1.docx]

**Supplementary table**

Correlating the demographic data, clinical phenotypes, laboratory profile and drug therapies with the common echocardiographic features in the patients demonstrated significant or near significant associations as shown in **Table 1**.

Because descriptive analyses revealed that all patients with pericardial thickening had MetS and did not follow on AZA, all patients with pulmonary regurgitation did not present with mucocutaneous lesions and were not on CS and all patients with seizures manifested with aortic thickening and mitral thickening, the risk estimates for the aforementioned cardiac lesions coupled with these clinical presentations could not be calculated.

**Table 1: Highlights on the associations of demographics, clinical phenotypes, laboratory profile and drug therapy with the common echocardiographic features in lupus patients**

|  | **Demographic characters*** | **Clinical data*** | **Laboratory*** |
| --- | --- | --- | --- |
| **Pericardial thickening** |  | **MetS** p=.045  Risk estimate = NA#    **AZA** p=.033  Risk estimate = NA#    **§** **Cumulative HCQ doses**  800 (400-1400) vs 4800 (2400-16800)  p=.042 | **§** **TG**  214 (194-282) vs 95 (73.5-148.5)  p=.024    **§** **HDL**  28 (25-29) vs 51 (37.5-65)  p=.004 |
| **Pericardial effusion** | **§** **Age at diagnosis**  38 (35-42) vs 23 (19-33)  p=.043 | **MetS** p=.008  (Risk estimate =14 CI 95% 1.55 – 126.57)    **Anti-HTNs** p=.053  (Risk estimate =5.5 CI 95% 1– 30.11)    **AVN** p=.046  (Risk estimate =16.67 CI 95% 1.3 – 212.5)    **§ Systolic BP**  130 (120-150) vs 110 (110-120) p=.013    **§ Diastolic BP**  90 vs 80 (70-90) p=.034    **§ Cumulative HCQ doses**  800 (400-2000) vs 4800 (2400- 14400)  p=.018 | **Proteinuria** p=.059  (Risk estimate =7.29  CI 95% 0.833- 63.79)    **§** **TG**  214 (174- 350) vs 95 (73-147)  p=.03 |
| **Pulmonary artery hypertension** |  |  | **§ LDL**  55 (49-75) vs 85 (69-118)  p=.046 |
| **Mitral thickening** |  | **Seizures** p=.032  Risk estimate = NA#    **Cognitive impairment** p=.033  (Risk estimate =6.29 CI 95% 1.27 – 31.1)    **Mucosal ulcers** p=.083  (Risk estimate =0.292 CI 95% 0.069 – 1.24)    **Arthritis** p=.079  (Risk estimate =0.153 CI 95% 0.018 – 1.29) |  |
| **Mitral regurgitation** |  | **Cognitive impairment** p=.014  (Risk estimate =7.93 CI 95% 1.43 – 44.03)    **Mucosal ulcers** p=.006  (Risk estimate =0.187 CI 95% 0.056 – 0.623)    **Malar rash** p=.008  (Risk estimate =0.208 CI 95% 0.063 – 0.692)    **Overall mucocutaneous disease** p=.019  (Risk estimate =0.259 CI 95% 0.082 – 0.822) |  |
| **Aortic thickening** |  | **Seizures** p=.016  Risk estimate = NA#    **§** **Cumulative CS doses**  2.5 (0 -155) vs 300 (40-1440)  p=.019    **CS** p=.049  (Risk estimate =0.205 CI 95% 0.043 – 0.98) | **§** **HDL**  39 (25 -50.5) vs 51 (37 -70)  p=.023 |
| **Aortic regurgitation** |  |  | **Pyuria** p=.035  (Risk estimate =12.6  CI 95% 1.183 – 134.24) |
| **Tricuspid regurgitation** | **§** **Current age**  35 (27 -43) vs 26 (22- 37.5)  p=.026 | **Malar rash** p=.063  (Risk estimate =0.341 CI 95% 0.108 – 1.8)    **CS** p=.053  (Risk estimate =0.323 CI 95% 0.1 – 1.036) |  |
| **Pulmonary regurgitation** |  | **Overall mucocutaneous disease** p=.066  Risk estimate = NA#    **CS** p=.025  Risk estimate = NA# |  |
| * Bivariate analysis by Chi square test, Fisher exact test or Mann- Whitney U test as appropriated. Risk estimate is calculated for categorical variables, NA#; risk estimate is non-applicable if a number of patients with or without the specified involvement in the cross tabulation equals zero, §; median (Interquartile ranges) of the involved vs non-involved group, MetS; metabolic syndrome, AZA; azathioprine, TG; triglycerides, HDL; high density lipoprotein, Anti-HTNs; anti-hypertensive drugs, AVN; avascular necrosis, BP; blood pressure, HCQ; hydroxychloroquine, LDL; low density lipoprotein, CS; corticosteroids. | | | |
